# Affordability Assessment from a Static to Dynamic Concept: A Scenario-Based Assessment of Cardiovascular Medicines

**DOI:** 10.3390/ijerph17051710

**Published:** 2020-03-05

**Authors:** Leila Zarei, Iman Karimzadeh, Najmeh Moradi, Payam Peymani, Sara Asadi, Zaheer-Ud-Din Babar

**Affiliations:** 1 Pharmacoeconomics and Pharma Management, Health Policy Research Center, Institute of Heath, Shiraz University of Medical Sciences, Shiraz, Fars, Iran; Leilazarei89@gmail.com; 2 Clinical Pharmacy, School of Pharmacy, Shiraz University of Medical Sciences, Shiraz, Fars, Iran; Karimzadehiman@yahoo.com; 3 Pharmacoeconomics and Pharma Management, Health Management and Economics Research Centre, Iran University of Medical Sciences, Tehran, Iran; 4 Pharmacoepidemiology, Health Policy Research Center, Institute of Heath, Shiraz University of Medical Sciences, Shiraz, Fars, Iran; peymani.payam@gmail.com; 5 Department of Clinical Pharmacology & Toxicology, University Hospital Zurich, 8091 Zurich, Switzerland; 6 Clinical Pharmacy, Shiraz University of Medical Sciences, Shiraz, Fars, Iran; sarah.asadi70@yahoo.com; 7 Medicines and Healthcare, Department of Pharmacy, University of Huddersfield, Queensgate, HD1 3DH Huddersfield, UK; Z.Babar@hud.ac.uk

**Keywords:** cardiovascular disease, affordability, scenario-based assessment, medication cost, middle-income countries, health care system

## Abstract

The out-of-pocket payments for prescription medications can impose a financial burden on patients from low- and middle- incomes and who suffer from chronic diseases. The present study aims at evaluating the affordability of cardiovascular disease (CVD) medication in Iran. This includes measuring affordability through World Health Organization/Health Action International (WHO/HAI) methodology. In this method, affordability is characterized as the number of days’ wages of the lowest-paid unskilled government worker. The different medication therapy scenarios are defined in mono-and combination therapy approaches. This method adds on to WHO/HAI methodology to discover new approaches to affordability assessments. The results show the differences in the medicines affordability when different approaches are used in mono-and combination therapy between 6 main sub-therapeutic groups of CVD. It indicates the medicine affordability is not a static concept and it changes dynamically between CVD therapeutic subgroups when it used alone or in combination with other medicines regarding patients’ characteristics and medical conditions. Hypertension and anti-arrhythmia therapeutic groups had the most non-affordability and hyperlipidemia had the most affordable medicines. Therefore, affordability can be considered as a dynamic concept, which not only affected by the medicine price but significantly affected by a patient’s characteristics, the number of medical conditions, and insurance coverage.

## 1. Introduction

Cardiovascular disease (CVD) is responsible for more than 12% of the global disease burden and this burden continues to rise [1,2]. Note that mortality from CVD has increased since 2007 worldwide [3], and approximately 25 million people in the world, who suffer from CVDs, will die due to this disease in the first half of the 21st century [4]. Previous evidence has shown that reducing the risk of chronic diseases in low- and middle-income countries (LMICs) can avoid loss of economic output associated with chronic diseases like CVDs [5].

It is estimated that CVDs have 38% of non-communicated diseases (NCDs)-related deaths [6]. According to the institute for health metrics and evaluation (IHME), CVDs are the most common cause of death globally, and the first cause of premature death in Iran [7]. Also, the global burden of disease’s (GBD) previous reports in 2010 and 2015 reveal that about a million disability-adjusted life years (DALYs) is caused by CVDs, which result in 20% to 23% of the burden of disease and it is regarded as the reason for death of almost half (46%) of Iranians. Moreover, the two leading reasons for death and DALYs in Iran, respectively, are ischemic heart disease and stroke [8]. In addition, based on the GBD 2015, Iran, with more than 9000 cases of CVD per 100,000 persons, is one of the countries with the highest CVD rates in the world [9].

The rising burden of CVDs will be especially severe in Iran, where the resources for treatment are already stretched to the limit; consequently, devising and applying some strategies so as to prevent and control the disease and its risk factors are on top of the Ministry of Health’s agenda over the past few years [8]. Likewise, there is a challenge—an apparently uncontrolled rise in healthcare expenses among Iranians. Although the total expenditure index has increased 30 times over the past 20 years, there was growth up to 71 times in the health sector, which is promptly reaching 10 percent of the gross domestic product (GDP) [10,11]. This will have at least two consequences; (1) increasing out-of-pocket (OOP) payments, (2) making treatments non-affordable, and increasing catastrophic health expenditures (CHEs) so that numerous people will refuse to request vital health care services, causing damage to their health statuses [11]. It is in this manner that figuring out how to make prescription medicines, and broadly speaking, health care, affordable has become a socioeconomic obligatory [12].

Affordability as a dimension of accessibility reflects the economic capacity for people to spend resources to use appropriate services [13]. Nowadays, there is a special focus on the accessibility and affordability of medicines in different international programs such as Millennium Development Goals (MDGs), Sustainable Development Goals (SDGs), and WHO health system building blocks [14]. On the other hand, the treatment of CVDs is highly dependent on medications [15], and access to good-quality and affordable CVDs drugs was considered in some previous studies [16,17,18,19,20]. In addition, there is an expanded focus on assuring that individuals are secure against financial risks related to the accessing of healthcare, especially in LMICs [21], because households in LMICs bear a significant financial burden, considering that each one of the two government funding and health insurance contributions is inadequate to address the healthcare needs of households. Accordingly, a significant proportion of healthcare payments are paid through OOP [22]. In Iran, people pay over 50% of the health expenses, while it is less than 30% in many countries [10]. What matters is that medicines usually compose a significant percentage of these OOP [23]. For this reason, affordability of health care, and especially medicines, at the micro and macro levels, has been raised in many LMICs by the three prominent affordability measurement methods, including the impoverishment, catastrophic payment, and World Health Organization/Health Action International (WHO/HAI) method [24,25,26,27,28,29].

The WHO/HAI method, as the common approach that measures affordability by using the salary of the lowest wage unskilled worker, has been used in various studies across LMICs [28,30,31,32,33,34,35]. Previous studies have pointed to some limitations of this method, including not-accurate estimations of affordability, given that numerous private workers have less income compared to government workers; not reporting non-optional costs; seasonal variation earnings; how many individuals’ lives depend on this earning; and other treatment expenses like consultations and diagnostics [36].

### The Rationale of the Study

The previous studies have emphasized on the enormous split between the accessibility and affordability of essential medicines for chronic diseases in LMICs and the need to improve affordability through developing pharmaceutical pricing policy, which advocates price cuts [28,37]. Khatib et al. investigated the details regarding accessibility and cost of CVDs medicines (aspirin, β blockers, angiotensin-converting enzyme inhibitors, and statins) in 18 countries, which were involved in the Prospective Urban Rural Epidemiology (PURE) study. Medicines were viewed as affordable as long as their consolidated expense was under 20% of households’ capacity-to-pay. The results indicated the inadequate accessibility and affordability of CVDs for hypertension and the secondary prevention of CVDs [38].

In other global studies, Maaike et al. examined the accessibility, pricing, and affordability of CVDs medications (including atenolol, captopril, hydrochlorothiazide, losartan, and nifedipine) in 38 developing countries through the standardized data that were gathered based on the WHO/HAI methodology. They claimed that in the public sector, the medicine expense was approximately 2.0 (lowest-priced generic) and 8.3 (originator brand) days’ earnings to buy one month of treatment with one of the CVDs’ medicines. At the point when nations were matched, though, the private sector was generally more expensive than the public sector. Atenolol was most affordable, with an average of 1.1 days’ salary for the lowest-priced generic. In addition, the lowest-priced generic was, in all cases, more affordable than the originator brand product, both in the public and private sectors. By and large, CVD medicines were the least affordable in low-income regions. Also, when upper–middle income countries contrasted with high-income regions, they scored especially well [39].

Vasheghani-Farahani et al. (2018a and 2018b) evaluated the affordability and improvement effects of medicines used for secondary prevention of CVDs (including aspirin, beta-blockers, angiotensin-converting enzyme inhibitors or angiotensin-II receptor blockers, and statins) in the Tehran province of Iran using WHO approaches. They stated that the treatment of CVDs with 21 selected lowest-price generic equivalent medicines was generally affordable, but the poorest households in Iran would be at risk of pushing under the poverty line because of the price of these CVD medicines [40,41]. Due to this fact that the CVD patients can still encounter a considerable expense, particularly with regards to multi-drug utilization [39,42], there is an interest to evaluate the impacts of health costs on households with the specific consideration of CVDs by multinational studies or single countries studies [38,43,44,45,46,47,48,49,50,51,52,53,54]. 

Affordability is a complex function of factors, and any explanation to the affordability problem will need to regard all of the related factors together. Current techniques for evaluating affordability in healthcare may be restricted by their attention to monotherapy and utilizing the defined daily dose (DDD) [48]. An increasingly compelling approach may include a more extensive perspective compared to the currently portrayed in the literature, so as to regard combination therapy as well as dose/schedule adjusted treatment in order to ensure healthcare services and medicines are actually affordable for patients. Due to the fact that a significant portion of health expenditures are pharmaceutical expenditures and considering the emphasis of the Iranian National Drug Policy (INDP) to improve the affordability as well as accessibility of drugs to patients who experience catastrophic healthcare expenditure due to major severe diseases, it is one of the reasons behind the development of a generic market promotion [41].

The present study aims at evaluating the affordability of CVDs medication on patients in the Iranian health system. In this study, we cover the literature gap regarding the current focus of affordability measurements on monotherapy and medicines in the WHO essential medicine list [29,36,55].

## 2. Materials and Methods 

Various techniques were employed to measure affordability. This includes measuring affordability by using the WHO/HAI methodology. The second was scenario development. The different medication therapy scenarios were defined in mono-and combination therapy approaches to assess affordability. 

### 2.1. Medicines Selection

In this cross-sectional study, we included all CVD’s medications that listed in 2017 Iran drug list (IDL) with different dosage form and dosages. The medicines were extracted from IDL using the Anatomical Therapeutic Chemical (ATC) Classification System, group C. Then, the affordability of CVDs medicines in different medication therapy scenarios was investigated, utilizing the standard treatment approach recommended by WHO/HAI as well as newly introduced scenario-based approaches regarding patients’ medical conditions and clinicians’ opinions. 

### 2.2. Affordability Measurement 

According to the WHO/HAI methodology, for chronic disease, affordability is characterized as the number of days’ wages of the lowest-paid unskilled government worker (LPGW) and expected to buy 30 days of treatment regimens. In the case of an acute condition, the treatment period is characterized as a full course of therapy. Paying over one day’s salary is considered unaffordable [37]. 

### 2.3. Scenario Development 

In this study, different medication therapy scenarios were defined in mono-and combination therapy approaches to assess affordability. This method adds to WHO/HAI methodology to discover new approaches to affordability assessments [36]. 

In this method, for calculating the number of needed unit doses under a treatment schedule, DDD-average maintenance dose per day for a medicine used for its main indication in adults is referenced and multiplied by a course of treatment. Therapeutic doses on CVD management vary by individual patients and medicines are prescribed according to their characteristics, medical history, and conditions, and DDD does not necessarily correspond to the recommended or prescribed daily dose (PDD) [56]. So, with the aim of avoiding patients missing a less/more standard daily dose in a real-life setting according to clinical guidelines such as American College of Cardiology/American Heart Association (ACC/AHA) [57,58], minimum and maximum recommended therapeutic daily dosages in monotherapy are determined. On the other hand, monotherapy and increasing the dose is not a preferred option due to increased risk and side effects, and clinicians choose combination therapy as a superior better-option in disease management. For example, in most hypertensive patients, guidelines support the use of drug combinations, and recently, treatment initiation with two drugs has also been recommended [58]. Therefore, according to clinicians’ opinions in combination therapy, regarding patients’ characteristics such as weight, age, and medical history and disease severity and comorbidities, combination therapy that includes different dose/schedule adjusted treatments was developed.

### 2.4. CVD Medicines and Therapeutic Group Selection 

Based on the ATC code, 223 medicines in group C or cardiovascular were registered in IDL, up to 2017. All of them were evaluated in this study and classified into six main therapeutic subgroups of CVDs (acute coronary syndrome (ACS), angina, cardiac arrhythmias, heart failure (HF), hypertension (HTN), hyperlipidemia) based on the last edition of the Applied Therapeutic: The Clinical Use of Drugs textbook and AHA/ACA guidelines.

### 2.5. Data Collection and Analysis

Needed data was collected for all CVD medications that were registered in IDL in 2017, from valid resources to calculate the number of days’ wages for a 1-month course of treatment. The details of data collection and affordability assessment was shown in the Appendix A. It included the ATC code, dosage forms, dose, the length of medicine use (for acute or chronic conditions), and the age group targeted by the medication, availability of the medication, medicine price, minimum daily salary of an unskilled worker, insurance coverage, and the DDD. If the patient had to spend more than one day’s wage on one day of LPGW, the medication therapy was labeled as non-affordable. The LPGW earned 8.8 US dollar per day, which is based on the exchange rate provided by the Iran Central Bank at the time of analysis (IRR 42,000 = USD 1).

### 2.6. Ethical Issues

The study was approved by the Ethics Committee of the Shiraz University of Medical Sciences. The consent form was not applicable for this study. The article does not require any human/animal subjects to acquire such approval.

### 2.7. Availability of Data and Materials

All data is available and can be provided by the corresponding author upon rational request. 

## 3. Results

### 3.1. General Review 

In total, 223 CVDs medicines registered in IDL were analyzed. Approximately, they constitute 7.4% of total registered medicines in IDL up to 2017. To assess their availability at the national level, the sales data on CVDs medicines were checked in Iranian pharmaceutical market sales data at the referenced year (2017). If there were no sales data on the specific medicine at the reference year, its market availability was investigated in Iranian pharmaceutical market sales data 3 years earlier; the medicines with no sales data during these four years were excluded from the assessment.

### 3.2. Scenario-Based Assessment 

To assess affordability, different medication therapy scenarios were defined in mono- and combination therapy approaches. The results were varied regarding the scenario type, indication, and patients’ condition. The details of each scenario are as follows.

#### 3.2.1. Monotherapy Approach

**Figure 1:**
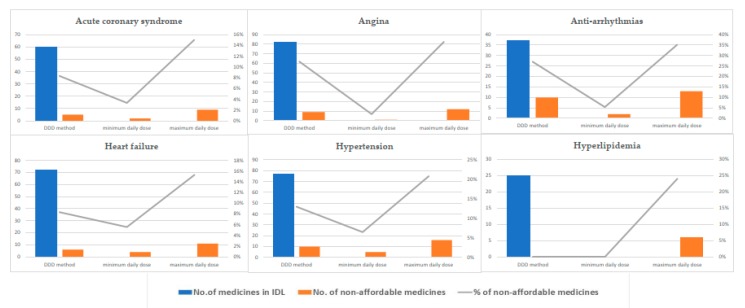
The affordability of medicines in cardiovascular disease (CVD) therapeutic groups based on monotherapy treatment scenarios.

In monotherapy, three treatment scenarios were designed, including standard treatment based on DDD, minimum, and maximum prescribed (recommended) daily dose. In these scenarios, CVD medicines were assessed with approaching in 3 major questions in monotherapy treatment: First, which therapeutic indication had the highest share of non-affordable medicines; second, which medicine had the highest affordability ratio; and third, what is the role of insurance coverage in the patients’ protection from financial risk. Figure 1 shows the affordability of medicines in CVD therapeutic groups based on these treatment scenarios.

The number of non-affordable medicines in each therapeutic group varies regarding the monotherapy scenario and increased by switching from minimum PDD dose to DDD and maximum PDD. However, anti-arrhythmias and HTN therapeutic groups have the highest percent of non-affordable medicines in all monotherapy scenarios, whereas in the hyperlipidemia therapeutic group, all medicines, except those in the maximum PDD scenario, were affordable.

**Figure 2:**
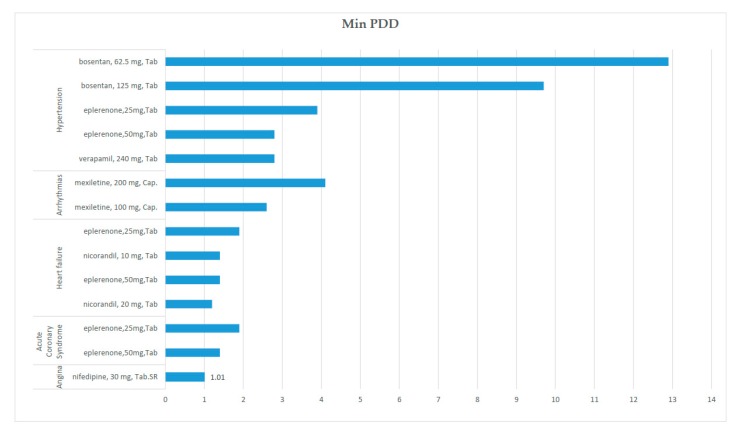
The non-affordable medicines in the minimum prescribed daily dose (PDD) scenario.

**Figure 3:**
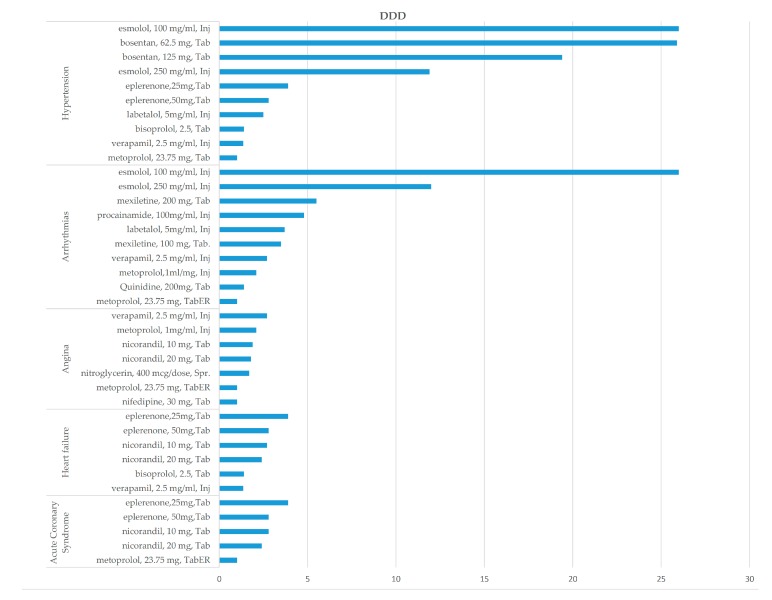
The non-affordable medicines in the defined daily dose (DDD) scenario.

**Figure 4:**
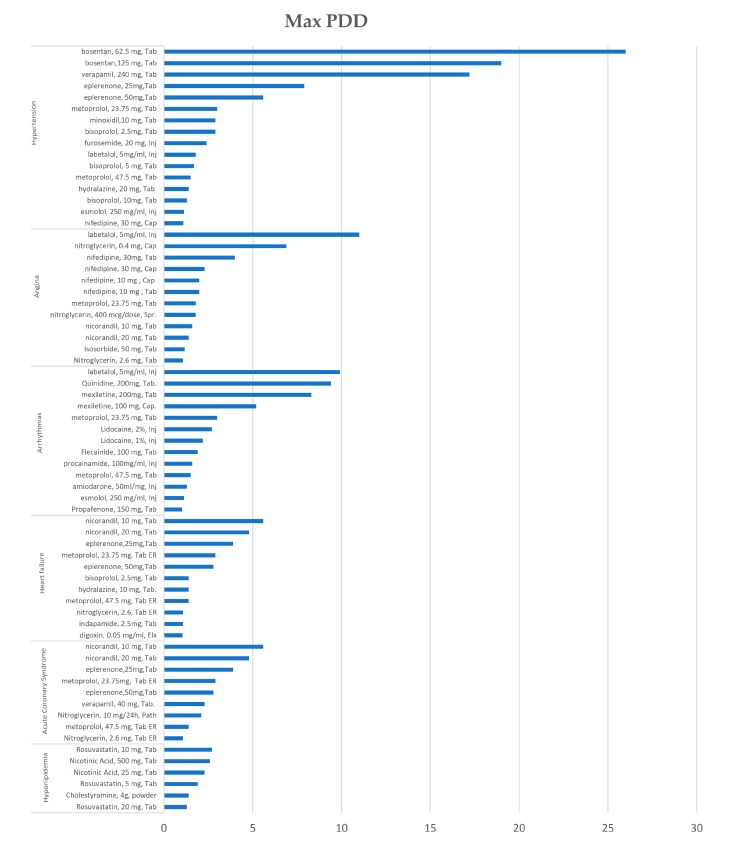
The non-affordable medicines in the maximum PDD scenario.

Figure 2, Figure 3 and Figure 4 show the most non-affordable medicines in each indication in monotherapy scenarios. The affordability ratio of each medicine is changed by monotherapy scenario, therapeutic indication, dosage, and dosage form. Based on minimum PDD, the highest affordability ratio belongs to bosentan (HTN), mexiletine (Anti-AR), and eplerenone (HF, ACS). In this scenario, all medicines in hyperlipidemia and angina (except nifedipine with an affordability ratio of 1.01) were affordable.

In the DDD scenario, esmolol and bosentan (HTN), esmolol and mexiletine (Anti-AR), and eplerenone (HF, ACS) were the most non-affordable medicines, with affordability ratios greater than 5.

Finally, in maximum PDD, bosntan, verapamil, and eplerenone (HTN), esmolol and mexiletine (anti-AR), were the most non-affordable medicines, with their affordability ratios greater than 5.

Generally, with considering scenario type, esmolol (Inj), bosentan (Tab), esmolol, 250 mg/mL, mexiletine (Tab), verapamil (Tab), labetalol (Inj), quinidine (Tab), eplerenone (Tab), nitroglycerin (Cap), nicorandil (TaB) are the top 20% of non-affordable medicines among all, with affordability ratios ranging from 5.2 to 26 of LPGW.

In total, 80% of off non-affordable medicines had no insurance coverage. Interestingly, in some cases, some medicines such as mexiletine (Tab, Cap), procainamide (Inj) and verapamil (Inj) remained non-affordable despite 70% insurance coverage; it means insurance coverage does not necessarily secure patients from hardship. Thus, more consideration is needed to improve the financial protection of patients.

Another point is related to the daily recommended dose. If the DDD were used as a base, the affordability ratio is the same; this is while when the clinicians’ opinions were considered; there may be a prominent difference in the affordability of one medicine in two indications of cardiovascular disease. For instance, in minimum PDD, the affordability ratio of eplerenone, 25 mg Tab, in HTN and HF was 3.9 and 1.9, respectively. Another example is related to nicorandil 10 mg Tab, which had the affordability ratios of 1.6 and 5.6. in angina and ACs in maximum PDD, respectively. Similarly, the affordability ratio of labetalol, 5 mg/mL Inj in HTN and Angina is 1.8 and 11, respectively. 

Moreover, the dosage and dosage form affect affordability. For example, in HTN monotherapy, according to the DDD scenario, the affordability ratio of esmolol, 250 mg/mL injection, is 12, while for esmolol, 100 mg/mL injection, it is 26. Additionally, 19.5 and 25.9 were the affordability ratio of bosentan 125 and 62.5 mg Tab. Accordingly, in Angina, the affordability ratio of metoprolol, 23.75 mg, Tab is 1.02 while in metoprolol, 1 mg/mL Inj, it reached 2.1. 

**Figure 5:**
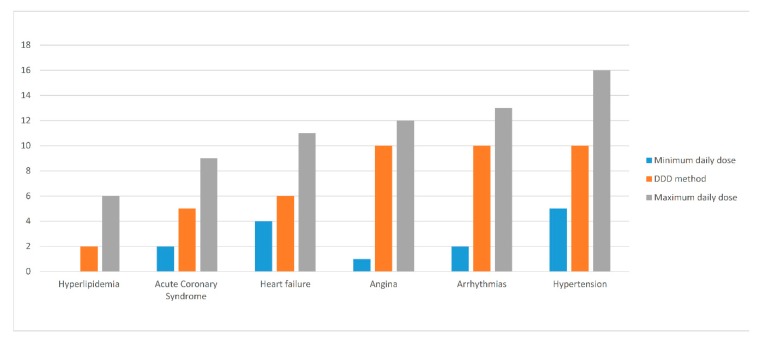
The non-affordable indications in monotherapy scenarios.

In total, this assessment shows (as seen in Figure 5) that regardless of the scenario type, HTN and hyperlipidemia therapeutic groups have the highest and lowest number of non-affordable medicines in CVDs mono- medication therapy. 

#### 3.2.2. Combination Therapy Approach

**Figure A1:**
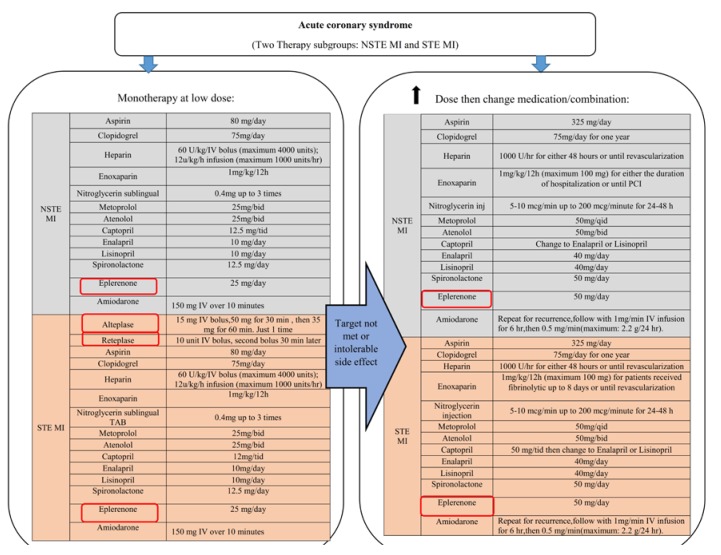
The different medication therapy scenarios in acute coronary syndrome (ACS).

**Figure A2:**
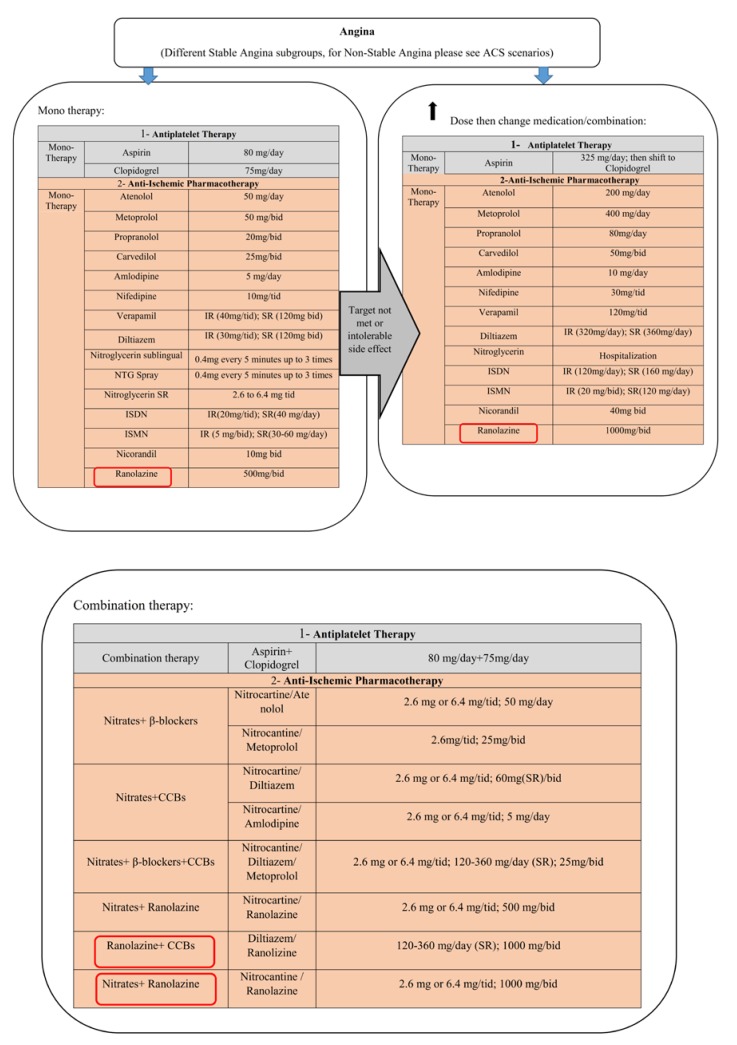
The different medication therapy scenarios in angina.

**Figure A3:**
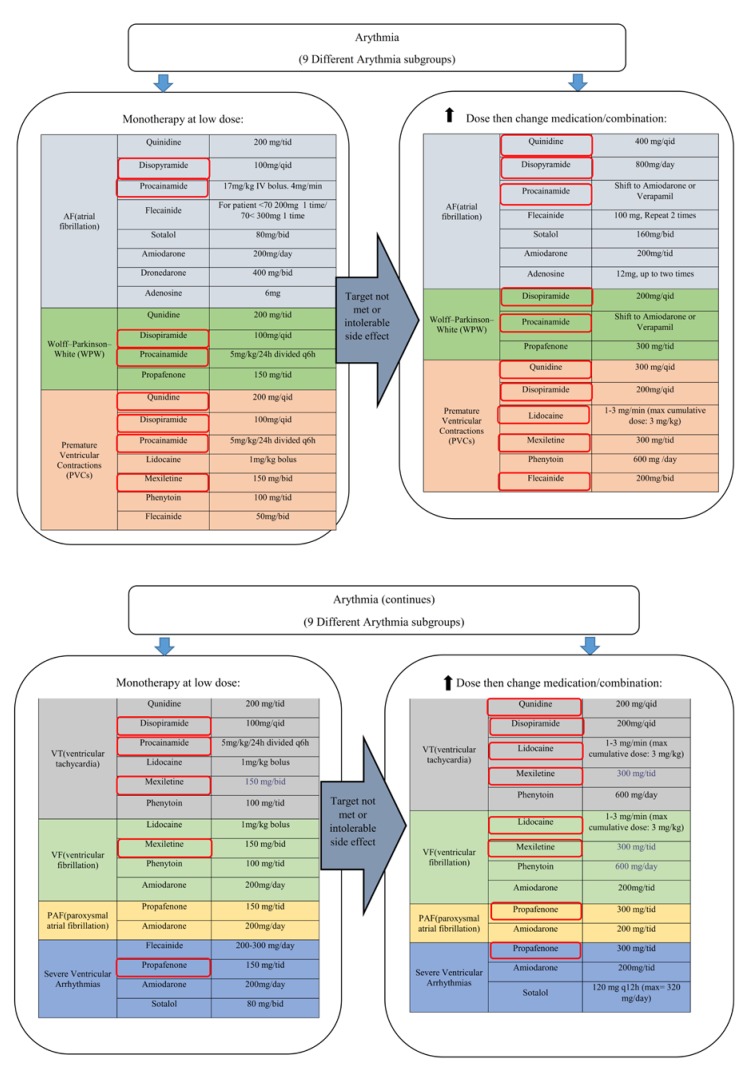

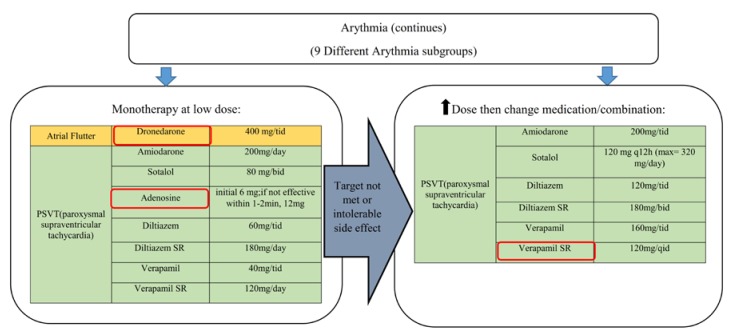
The different medication therapy scenarios in arythmia.

**Figure A4:**
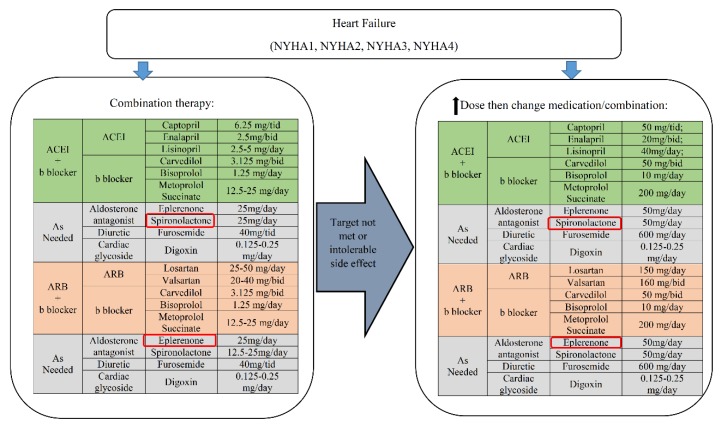
The different medication therapy scenarios in heart failure.

**Figure A5:**
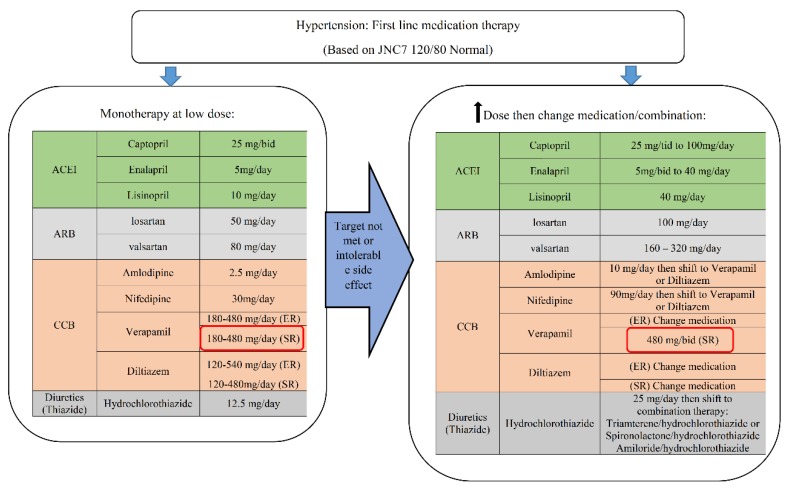
The different medication therapy scenarios in hypertension.

**Figure A6:**
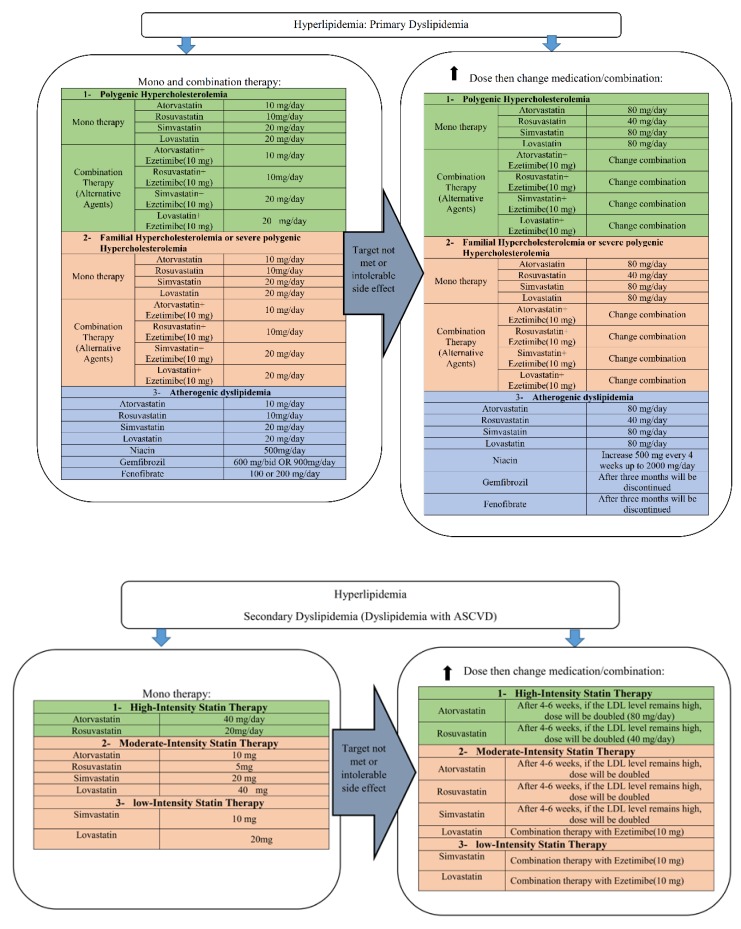
The different medication therapy scenarios in hyperlipidemia.

When the clinical targets fail in monotherapy, there are two treatment options: increasing the dose or combination therapy. The affordability assessment of increasing dose was considered in the maximum PDD in monotherapy scenario. However, an increase in dose is not a preferred option due to the increasing risk and side effects, and clinicians choose combination therapy as a superior better-option in disease management. So, regarding the patient’s characteristics such as weight, age, medical history, disease severity, and comorbidities, different dose/schedule adjusted treatments were defined. In this study, more than 304 dose/adjusted scenarios were defined according to clinicians’ opinions for assessment (Figure A1,Figure A2,Figure A3,Figure A4,Figure A5 and Figure A6). In total, affordability assessments in combination therapy have three possible results. 

Target medicines were

1- Affordable in both monotherapy and combination therapy.
2- Affordable in monotherapy and non-affordable in combination therapy
3- Non-affordable in both monotherapy and combination therapy

For example, in angina, stable and non-stable angina are two different conditions where different medicines may be prescribed. Metoprolol 50 mg/day is affordable in monotherapy. It still remains affordable when it prescribed in combination with other affordable medicine, i.e., nitroglycerin 2.6 and 6.4 mg, while amlodipine 5 mg/day and valsartan 80 mg/day switch from being affordable monotherapy to non-affordable therapy when prescribed in combination with each other. The third, nicorandil 20 mg/twice a day is non-affordable medicine in monotherapy and, in combination with diltiazem or amlodipine, constitutes non-affordable combination therapy. 

There are numerous examples that indicate the affordability has a dynamic nature and it changes when the treatment schedules change or modify regarding clinician opinions in mono-and combination therapy. So, the standard treatment based on DDD may not be appropriate when affordability at a micro patient level is the main aim.

## 4. Discussion

This study uses a novel approach to assess CVD medicine affordability. This scenario-based approach adds to WHO/HAI standard methodology. The clinician-based scenarios consider different treatment schedules for patients in different CVD conditions. 

To assess affordability, the WHO/HAI use the DDD approach as the current standard to estimate the amount of medicines needed during a course of treatment [36]. The best advantage of this standard method is the possibility of cross-country and international comparison; characterizing results in this way allows universal examinations of the level of affordability [59], but when decision-makers need a precise analysis of affordability in order to make a reliable policy, the national and regional analysis based on real-world practice is preferable [13,24]. For instance, when medicine affordability at the patient level is important, the DDD may not be appropriate. In the current study, to overcome this limitation alongside to standard approach, the dose/schedule adjusted medication therapy scenarios regarding individuals’ medical conditions and characteristics and clinical response to the treatment was introduced in mono-and combination therapy as an appropriate and logical option to have a rational picture of affordability of CVD medication therapy in the country.

Assessing the affordability of CVD medicines between 6 main sub-therapeutic groups has shown that affordability changes dynamically between therapeutic groups. The hypertension and anti-arrhythmia therapeutic groups have the most non-affordable medicines and hyperlipidemia has the most affordable medicines in Iran. The evidence shows poor accessibility and affordability of cardiovascular medicine in investigated countries. Ewen et al. conducted a secondary analysis of data from 30 surveys in LMICs, conducted from 2008 to 2015 using the WHO/HAI method. The results show 11.9% of non-affordable medicines in low-income countries belong to CVDs [60]. The Prospective Urban Rural Epidemiological (PURE) study showed low access and affordability of blood pressure-lowering medicine for the majority of the population in LMICS [38]. Mendis et al. showed the non-affordability of combination treatment for coronary heart disease between African countries with high variation in affordability ratios, from 18.4 days’ wages in Malawi to 1.5 in Sri Lanka [24]. Also, affordability assessment of essential CVDs medicines by Abdel Rida et al. (2019) in Qatar and Lebanon revealed that only the public sector in Qatar, the current situation of access to cardiovascular disease medicines in both countries needs key policy decisions to improve access [61].

Our study also shows the significant effect of different approaches to measuring medicines’ affordability. In the current assessment, the affordability changed based on the clinician’s opinion on the needed daily dose. The number of non-affordable medicines raised when treatments switch from minimum PDD to DDD and maximum PDD, which is rational due to an increase in the needed dosage. A notable finding in this research was having more than one affordability ratio for one medicine in two different therapeutic indications, such as eplerenone, 25 mg, tablet in hypertension and heart failure when it was prescribed in minimum daily dose. The affordability assessment is more complicated when the patient-adjusted treatment schedule shifts from monotherapy to combination therapy [62]. In this way, some of the affordable medicine were made non-affordable when it was used in combination with other CVD medicines such as amlodipine 5 mg/day in a different indication. It is expected for complicated patients, who need additional medicines, affordability would probably be magnified and worse than normal. Numerous health conditions coincide, subsequently, the accessibility and affordability issues would presumably be amplified [63].

In total, the observed differences indicating the nature of affordability in a dynamic setting is not static. The patients with different CVD conditions have different affordability. Moreover, the patients who need more or less recommended prescribed doses can afford more and less, respectively. It is worth mentioning, in all assessments where the LPGW measure was based, the applicability of LPGW measure in a policy context has its limitations such as overestimation of medicine affordability due to not considering the proportion of the population whose incomes are less than of LPGW, not accounting other costs including non-discretionary expenditures, income fluctuations as well as other treatment-related costs [59].

Yet, as the literature and our findings indicate, the effect of the methods selected for measuring affordability along with the thresholds chosen inside those methods is critical on final outcomes [13,64]. 

Experimental literature regarding affordability reflects the trouble of univocally understanding the idea of affordability and achieving an appropriate and general threshold for affordability [64]. In order to support governments to enhance access to medicines, we, in this manner, would like to discuss how to best to address the affordability question to promote transparency and intertemporal and global examination. We think it is time to revise and refine the affordability assessment approach in order to permit quantifying the intrinsically “dubious” idea of affordability. Also, we ask for an amend of the estimation of affordability, switching it from a static to a dynamic concept that closes it to real-world practice to expand the governments’ awareness and keep moving in order to act quickly on access issues [13]. 

## 5. Limitations of the Study

In monotherapy scenarios, we only investigated the affordability of cardiovascular medicines in category C based on WHO/ATC, while CVD patients may need multiple medications in another ATC system. For example, alteplase and reteplase, which are used for ACS indication are in B category. Such medicines may only be investigated in the dose/adjusted combination therapy scenarios and are not included in the monotherapy assessment. 

The costs and affordability patterns covered in this study also indicate that it could impact medicine adherence; however, this impact is not fully investigated in this study.

## 6. Conclusions

To the best of our knowledge, this is a first attempt to investigate the affordability of medication therapy in a specific disease in different treatment scenarios in Iran. The evidence regarding the affordability of medicine in CVD reveals that affordability is not a static concept. The current benchmark has some deficiency, and application of results based on this approach in local decision making, particularly in financial protection policies to achieve universal health coverage (UHC), needs more caution and considerations. So, revising and refining the affordability concept is strongly recommending to pharmaceutical policymakers.

## Appendix A

The following actions were performed to quantify needed medicines in treatment regimes in different CVDs therapeutics subgroups.

For monotherapy:

1. DDD of each medicine was obtained from the WHO Collaborating Centre for drug statistics methodology (http://www.whocc.no/atc_ddd_index/).
2. Considering the fact that the CVDs medicine dose is based on the patient’s characteristics and disease conditions, the exact dose cannot be defined the same for all patients with CVDs. Therefore, moreover DDD amount, the minimum and maximum daily dose of each medicines was defined according to the as American College of Cardiology/American Heart Association (ACC/AHA) [57,58] and pharmacotherapy reference books like Applied therapeutics.
3. The majority of patients with CVDs require lifelong treatment, these CVDs medicines were considered as chronic disease medicines and the calculations were performed based on a treatment period of 1 month. In the case of acute condition, the treatment period was characterized as a full course of therapy.
4. The amount of out of pocket payment by patients for each medicine, was calculated regarding the amount of insurance coverage and extra financial protection such as MOU between Iran FDA and health insurance organizations.
5. The treatment cost was calculated according to weighted average of medicines’ prices.

## Appendix B

The Figure A1,Figure A2,Figure A3,Figure A4,Figure A5 and Figure A6 show the different medication therapy scenarios in CVDs thereputic subgroup including acute coronary syndrome (ACS), angina, cardiac arrhythmias, heart failure (HF), hypertension (HTN), hyperlipidemia, respectively.
